# Frailty and the risk of cognitive impairment

**DOI:** 10.1186/s13195-015-0140-3

**Published:** 2015-08-03

**Authors:** Samuel D. Searle, Kenneth Rockwood

**Affiliations:** Department of Medicine, Dalhousie University, 1421-5955 Veterans’ Memorial Lane, Halifax, NS B3H 2E1 Canada; Capital District Health Authority, 1421-5955 Veterans’ Memorial Lane, Halifax, NS B3H 2E1 Canada; Centre for Health Care of Elderly, Division of Geriatric Medicine QEII Health Sciences Centre, Capital District Health Authority, 1421-5955 Veterans’ Memorial Lane, Halifax, NS B3H 2E1 Canada

## Abstract

Aging occurs as a series of small steps, first causing cellular damage and then affecting tissues and organs. This is also true in the brain. Frailty, a state of increased risk due to accelerated deficit accumulation, is robustly a risk factor for cognitive impairment. Community-based autopsy studies show that frail individuals have brains that show multiple deficits without necessarily demonstrating cognitive impairment. These facts cast a new light on the growing number of risk factors for cognitive impairment, suggesting that, on a population basis, most health deficits can be associated with late-life cognitive impairment. The systems mechanism by which things that are bad for the body are likely to be bad for the brain can be understood like this: the burden of health deficits anywhere indicates impaired ability to withstand or repair endogenous and environmental damage. This in turn makes additional damage more likely. If true, this suggests that a life course approach to preventing cognitive impairment is desirable. Furthermore, conducting studies in highly selected, younger, healthier individuals to provide ‘proof of concept’ information is now common. This strategy might exclude the very circumstances that are required for disease expression in the people in whom dementia chiefly occurs (that is, older adults who are often in poor health).

## Introduction

Until death intervenes, aging in humans is inevitable and inexorable. The aging process has been conceptualized as occurring in small increments as a result of a preference for resources that serve reproduction over those that serve repair. With time, such microscopic damage accumulates, leading to clinically detectable deficits, which themselves manifest as tissue, organ, and functional impairment [1].

Longevity, contrary to initial predictions, has been accelerating, in part because health care continues to improve in treating disability and comorbidity [2]. How this will play out is as yet unclear. Even given recent revisions in whether late-life mortality plateaus occur [3], there is concern that the burden of chronic disease might increase, as people more often survive illnesses that are now more disabling than fatal. Alternately, although more diseases might accumulate, better management could result in a lesser overall burden on health. This in turn would result in more chronic illness, even if individual diseases themselves were less burdensome. The evidence to date favors the latter, in part because older adults who are disabled are more likely to die than their non-disabled age peers [2, 4]. Still, given how tightly age is linked to dementia risk, the worry persists that increasing late-life longevity, to the extent that it drives population aging, will fuel a rising number, and proportion, of people with dementia [5].

Although understanding health and aging requires some nuance, the starting point is clear. At least since Gompertz in the 19th century, we have recognized that across the adult lifespan the risk of death increases exponentially with age. One implication of this is that, although single-system illness predominates in mortality risk when people are younger, the acceleration in mortality risk beginning partway through the sixth decade reflects that many interacting factors are implicated in causing death [6]. Equally clear, however, is that not every one of the same age has the same risk of death at that age. For some time, the term applied to the increased risk of death at a given age, compared with their age peers, has been frailty [7]. Likewise, people who are fit have a lower risk of death than do others of the same age.

Frailty is now well recognized as a risk factor for dementia [8–10]. Even so, nuance is required here too. In general, two views of frailty are widely recognized [11] and they suggest different mechanistic understandings. By way of disclosure, we are proponents of viewing frailty as reflecting deficit accumulation (that is, of being more a state than it is a phenotype or syndrome), which is a competing (if still complementary) view.

Our goals here are, first, to critically evaluate the claim that frailty is related to cognitive impairment and, second, to suggest implications of this relationship for understanding dementia prevention and treatment and for the design and analysis of clinical trials.

## Methods

A PubMed review was performed (and last updated 4 Nov. 2014) by using the key words ‘frail’, ‘frailty’, ‘frail elderly’, ‘cognitive impairment’, ‘dementia’, and ‘Alzheimer’s disease’ and limiting publication date to 1 Jan. 2000 up to and including the above date. Initially, we identified 1145 articles, which then were screened initially by abstract and where necessary by manuscript, resulting in 317 articles that met our criteria. The vast majority of these articles identified ‘frail’ as individuals at increased risk of adverse outcome. Thirty-nine articles presented associations or incident risks of between an operationalized frailty assessment and cognitive impairment; two specifically identified cognitive impairment as a risk factor for incident frailty [9, 12]. Where frailty was operationalized, the majority used a variation of the phenotype definition [13]. Additional hand searches were performed, yielding 30 articles that were not found in the organized search, because they covered either frailty or cognitive impairment (in isolation).

## Frailty as a phenotype and frailty as deficit accumulation

The phenotypic approach to frailty is widely used [13]. It holds that frailty is best understood as a syndrome. Five features are proposed: impaired grip strength, exhaustion, slowed gait speed, weight loss, and reduction in activities. An early report reckoned that the presence of frailty increased the risk of dementia. This was of interest and has motivated a great deal of work (in part to address the issue of whether the frailty syndrome should be expanded to include aspects of cognition and affect) [14].

Given that each of the items that make up the frailty phenotype is recognized as a risk factor for dementia [8, 15, 16], that they should also convey risk when combined cannot be seen as surprising. In consequence, here we will evaluate more the relationship between health deficits broadly construed—as potentially including, but not being restricted to, the five phenotypic features. That is to say that we will focus on another common view of frailty, which is that it is a state of increased risk; this risk arises in relation to the number of health deficits that people have and is mitigated by protective factors [17–19]. According to this formulation, the reason that as people age they are more likely to die and that people of the same age have varying risks of death is that, in general, the risk of death is related to the number of health deficits that people accumulate. In short, the more health deficits that an individual has, the more likely they are to die or to experience other adverse health outcomes, including worsening health status. Not everyone accumulates deficits at the same rate, and it is the people who have accumulated the most deficits who, at any age, are more likely to die than their age peers. This then is the basis of frailty [20].

The deficit accumulation approach yields several important features [21]. In cross-sectional evaluations at least from age 50 (and, in some Western studies, across the life course) in high-, middle-, and low-income countries around the world [21–24], health deficits accumulate at approximately the same rate (about 3.5 % per year) and are typically higher in women than in men. Consistent across frailty indexes (FIs), there appears to be a fixed limit to deficit accumulation. The deficit accumulation approach operationalizes frailty as the proportion of things wrong (that is, as the ratio of the number of health deficits present in an individual to the number of health deficits that were considered). For example, in a database that included 50 items that met the criteria to be considered health deficits, a person in whom 10 such deficits were present would have an FI of 10 out of 50, or 0.20. As it turns out, in both community-dwelling and hospitalized patients (and in intensive care unit series), the 99 % limit to frailty is 0.7. In short, at least 99 % of people will have FIs of less than 0.7 [22, 23, 25]. That is because the closer an individual is to an FI of 0.7, the greater is their risk of dying.

Health deficit accumulation begins as a consequence of subcellular processes [1]. How subcellular damage scales up to produce clinically detectable health deficits is a matter of ongoing inquiry [26]. Of note, a key step appears to be captured by subclinical events; for example, even minor laboratory abnormalities can be detected in otherwise well people, and their presence increases the risk of adverse health outcomes [27].

Inevitably, the brain is not spared in the aging process. Both cognitive impairment and dementia, in their various forms, rise with age [28]. By this line of reasoning, it is no coincidence that Alzheimer’s disease incidence is highest in those who are at least 80 years old because these will be the people with the greatest number of deficits otherwise. Recent data suggest that deficit accumulation and cognitive impairment are related, regardless of whether the deficits are considered as traditional risk factors [10, 29, 30].

## Aging: health deficits accumulate in the body and brain

In the Rush Memory and Aging Project, older adults who were frail demonstrated more Alzheimer’s pathology than did people who were not frail. Of some importance, this was true not just in people with dementia but in the non-demented patients as well [31]. In other words, physical frailty in the body reflected the accumulation of neuropathological lesions in the brain more than it did cognitive function. Interestingly, there seemed to be no relationship between frailty and microinfarcts or Lewy body findings in this particular study. This result has been replicated in data from the Religious Orders Study. More recently, published neuropathology data have shown additional links between cognitive impairment and frailty, suggesting common mechanisms [9, 32]. The full picture is still murky. As highlighted in a recent review, longitudinal studies have identified frailty to be a risk factor for non-Alzheimer’s dementia as well as more general cognitive impairment [8]. Note, however, that clinical dementia was not present in a significant proportion of the patients who met neuropathological criteria for Alzheimer disease; why this is so remains unclear, although speculation casts doubt on whether it is amyloid plaques or traditionally less readily demonstrable amyloid protein forms that are associated with neurotoxicity [33]. Abnormal amyloid deposition appears in some pathways to be necessary but not always sufficient and sometimes to be irrelevant. In short, it might very well be that amyloid deposition is more a risk factor than a cause. Furthermore, it might be that combinations of not just clinical but neuropathological deficits are needed: for example, work from the Honolulu-Asia Aging Study showed that multiple pathologies were associated with dementia, including in people with Alzheimer’s disease [34].

In short, it appears that as a range of health deficits accumulate, more disease entities will appear. This is not restricted to dementia but rather is highlighted in recent publications which show that so-called ‘non-traditional risk factors’ increase the risk for other late-life health problems, including osteoporosis [35] and cardiovascular disease [36]. That many outcomes are multiply determined is the challenge of studying the diseases of aging [4]. A sharp focus on disease mechanisms, studied in their purest form, has undergirded considerable scientific advances, and so it is not surprising that this is the dominant approach in Alzheimer disease. This emphasis on studying ‘pure’ Alzheimer’s disease extends into clinical trials, which commonly focus on patients who have little else wrong. If multiple deficits are required for disease expression in late life, how wise a strategy this is remains unclear. Although Alzheimer’s disease can tragically be seen in young people and in older adults who are otherwise fit, the list of individual risk factors that are associated with it is long, including ischemic cardiovascular disease, cardiac arrhythmias (notably, atrial fibrillation), congestive heart failure, atherosclerosis, hypertension, chronic kidney disease (CKD), insulin insensitivity, sleep disorders, chronic inflammation, immunosenescence, and obesity [8, 37].

## Multifactorial mechanisms of cognitive impairment

As noted in a recent *Nature* commentary, ‘[t]he problems of old age come as a package’ [4].

Many of the comorbidities investigated as risk factors for dementia themselves are associated with additional risks for cognitive impairment. For instance, CKD, most commonly caused by diabetes mellitus and hypertension in the developed world, has been shown to be a risk factor for cognitive impairment [38]. Unsurprisingly, CKD is most prevalent at older ages. Common mechanisms have been considered: both the brain and kidneys are uniquely profused and sensitive to microvascular injury, and so perhaps cognitive impairment in people with CKD represents the same process in different organ systems. In our view, however, this cannot be the complete story of their coincidence. CKD exacerbates hypertension, limits medication choices in patients with diabetes, and contributes to a range of metabolic abnormalities and complications such as anemia, acidosis, hyperphosphatemia, hypoalbuminemia, and hyperparathyroidism. These are themselves related to cognitive impairment and not solely via microvascular mechanisms. For example, anemia has been shown to increase risk for Alzheimer’s disease [39], and even mild anemia has been associated with poorer performance on Trails B testing [39]. Likewise, changes that synergistically worsen renal sodium and water physiology are also common in older adults, especially those who are frail. Hyponatremia is common in older adults and has been proposed to reflect the combined effects of a weaker central thirst response, abnormal antidiuretic hormone levels, and decreased ability of the kidneys to concentrate urine [40]. Sodium disturbances have been a potent stimulus for delirium [41, 42]. The many interacting mechanisms by which cognitive impairment can arise in the face of accumulating health deficits are not limited to CKD. Cognitive impairment is linked to congestive heart failure [43], again with many factors linking them beyond vascular risk factors. Decreased cerebral perfusion, cerebral reactivity [44], oxidative stress, inflammation, microemboli, and neurohumoral affects are each shared by the two conditions. Similarly, many other factors can be implicated in late life dementia, reflecting the variable pathways by which it can arise [10].

As with the individual items that make up the frailty phenotype, combining other known risk factors for cognitive impairment (here, vascular risk factors) improves prediction of dementia risk [45]. Strikingly, however, even health deficits not routinely identified as risk factors for dementia or cognitive impairment have been shown to be factors predicting dementia and Alzheimer’s disease [30]. This suggests that there are multiple mechanisms for developing dementia beyond vascular risk factors and that a systems approach might be useful in understanding the link between risk factors and late-life dementia.

## Deficit accumulation and failing repair processes

One example of a systems approach to understanding how multiple risk factors might combine to be associated with late-life dementia is the application of queuing theory to deficit accumulation. A broadly applicable discipline in applied mathematics, queuing theory describes how a queue operates [46], how it lengthens or shortens, and so provides an analogy with deficit accumulation. The length of a queue is a function of the rate at which people arrive at the queue and the amount of time it takes to process them. Likewise, the number of deficits that a person accumulates (that is, the magnitude of their FI) is the product of the rate at which damage arises and the rate at which it is repaired (or removed). With this model, the assumption is that, in the near term, the rate of damage arising from both the external and the internal environment is constant, so that change in the FI (usually increasing) chiefly reflects change (usually slowing) in the rate of repair/removal of damage. It is important to note that damage makes further damage more likely, in that as recovery time increases, there is less time to repair deficits before new damage arises. In consequence, health deficits accumulate, exponentially so, especially as repair processes themselves become damaged.

This has been recently proposed for dementia specifically [10]. It follows that improving both the environment (that is, the rate at which damage arises) as well as the medical management of health issues (for example, comorbidity management, improved social structure, and the like) should help both cognitive impairment and frailty by improving repair capability and shortening recovery times. Understanding dementia risk in relation to “impaired repair function” is to invoke what is purposely a broad construct. At a very general level, a deficit arises whenever damage goes unremoved or unrepaired [46]. In consequence, if the nature of damage varies—if the range of risks that are associated with dementia is wide—then one way of understanding how these diverse exposures result in dementia has less to do with the damage itself, than it does with either an aberrant repair process, or simply a slow one. On average, repair processes slow with time—recovery time increases with age [46]. Considering how commonly it occurs in late life, what gives rise to dementia may be less the nature of the damage than a more widespread increase in recovery time.

Brain deficits accumulate due to many mechanisms [26, 46, 47]. Consider that to date, the single most important intervention for both frailty and cognition appears to be exercise [48]. Exercise is an example of how a single intervention can have multiple mechanisms for mitigating the development of cognitive impairment. These include direct effects on brain function and structure by neurogenesis, angiogenesis, synaptogenesis, hippocampal volume, attenuated frontal loss of grey and white matter, and increased connectivity on the large scale brain circuitry [49]. This appears to be in addition to its indirect effects on cognitive functioning via treatment of cardiovascular risk factors, depression, anxiety, chronic stress, and potentially diet and sleep [48]. In short, just because a disease might arise from many mechanisms, mechanisms that could even be pragmatically unknowable for given individuals, interventions can still be possible, if such interventions are broadly based. Likewise, there is evidence that medical management of a range of comorbidities can improve cognitive outcomes even if the degree of improvement in relation to single maneuvers varies [49–51].

## Summary

Many comorbid physical illnesses are noncontroversially associated with changes in the brain. A large number of illnesses are associated with the classic lesions of Alzheimer’s disease. Intriguingly, in the Honolulu-Asia Aging Study and the Rush Memory and Aging Project, it has been demonstrated that the relationship between brain pathology and cognitive impairment is best understood in relation to the accumulation of lesions—atrophy, plaques, tangles, Lewy bodies, and microinfarction [34]—but in the latter study, this relation is not associated with any single lesion type.

Likewise, aging and cognitive impairment are closely related. This appears not to be by coincidence. With aging, cellular and molecular damage accumulates, ultimately giving rise to deficits that are visible by laboratory measures and as macroscopically detectable variables. These macroscopically detectable health deficits can have the status of disease. Common diseases, such as cardiovascular risk factors, now are noncontroversially associated with all causes of late-life cognitive impairment, including Alzheimer’s disease. Recent work has extended this approach to understanding late-life cognitive impairment to a large variety of items that traditionally have not been associated with dementia.

There might be particular merit, especially in diseases expressed chiefly in late life, of considering the malign influence of interacting factors, and not just single mechanisms, on three grounds. First, it appears to be generalizable, having been demonstrated further in the SHARE (Survey of Health, Ageing and Retirement in Europe) database [29]. Second, it likewise seems to extend not just to late-life cognitive impairment but also to other disorders, such as coronary heart disease events [36] and osteoporotic fractures [35]. Third, it allows an understanding of the proliferation of a very large number of putative risk factors for dementia—something that now risks exhaustion in not just the public but also the scientific community.

Understanding that frailty and cognitive impairment are linked has implications for how we test drugs for dementia. At present, many proof-of-concept studies with disease-modifying drugs are focusing further upstream: on healthier people who are typically younger and have few things wrong and even few symptoms. Perhaps, however, the impairment represented by deficit accumulation (a collective series of events that impair repair capacity generally) is what is needed to allow the earliest lesions seen in ‘Alzheimer’s’ to express their toxicity and so give rise to disease. If this is true, then only treatment effects will be found on those who are impaired. This intriguing observation is motivating further inquiries by our group.

## Conclusions

Frailty and cognition are related to each other and to aging (Box 1). The growing list of risk factors for dementia might simply reflect that both health deficit accumulation (frailty) and cognitive impairment are common in late life. Clinical trials in dementia should consider not excluding frail older adults, as frailty appears to drive disease expression and might be needed for the classic neuropathology of dementia to express its deleterious effects.

## Note

This article is part of a series on *The impact of acute and chronic medical disorders on accelerated cognitive decline*, edited by Carol Brayne and Daniel Davis. Other articles in this series can be found at http://alres.com/series/medicaldisorders

## Box 1 Highlighting points

- Frailty has been linked to cognitive impairment.
- Shared mechanisms might include both shared subcellular pathophysiology (for example, oxidative stress and protein misfolding) and systems mechanisms—as well as impaired repair (for example, failures in chaperone proteins, autophagy)—give rise to deficits at this level.
- These mechanisms are not unique to dementing illnesses and, especially in their multiplicity, implicate what is seen with aging.
- If the dementias, which occur chiefly in late life, exist because of multiple deficits, reflecting multiple mechanisms, this calls into question the notion of ‘proof of concept’ for Alzheimer’s disease treatment that focuses on younger patients with few health problems.

## Abbreviations

CKD: chronic kidney disease
FI: frailty index
